# Neural transcriptome reveals molecular mechanisms for temporal control of vocalization across multiple timescales

**DOI:** 10.1186/s12864-015-1577-2

**Published:** 2015-05-27

**Authors:** Ni Y Feng, Daniel J Fergus, Andrew H Bass

**Affiliations:** Department of Neurobiology and Behavior, Cornell University, 14853 Ithaca, NY USA; Current Address: North Carolina Museum of Natural Sciences, Genomics and Microbiology, 27601 Raleigh, NC USA

**Keywords:** Vocalization, Motoneuron, Transcriptome, Gene expression, Seasonal reproduction

## Abstract

**Background:**

Vocalization is a prominent social behavior among vertebrates, including in the midshipman fish, an established model for elucidating the neural basis of acoustic communication. Courtship vocalizations produced by territorial males are essential for reproductive success, vary over daily and seasonal cycles, and last up to hours per call. Vocalizations rely upon extreme synchrony and millisecond precision in the firing of a homogeneous population of motoneurons, the vocal motor nucleus (**VMN**). Although studies have identified neural mechanisms driving rapid, precise, and stable neuronal firing over long periods of calling, little is known about underlying genetic/molecular mechanisms.

**Results:**

We used RNA sequencing-based transcriptome analyses to compare patterns of gene expression in VMN to the surrounding hindbrain across three daily and seasonal time points of high and low sound production to identify candidate genes that underlie VMN’s intrinsic and network neuronal properties. Results from gene ontology enrichment, enzyme pathway mapping, and gene category-wide expression levels highlighted the importance of cellular respiration in VMN function, consistent with the high energetic demands of sustained vocal behavior. Functionally important candidate genes upregulated in the VMN, including at time points corresponding to high natural vocal activity, encode ion channels and neurotransmitter receptors, hormone receptors and biosynthetic enzymes, neuromodulators, aerobic respiration enzymes, and antioxidants. Quantitative PCR and RNA-seq expression levels for 28 genes were significantly correlated. Many candidate gene products regulate mechanisms of neuronal excitability, including those previously identified in VMN motoneurons, as well as novel ones that remain to be investigated. Supporting evidence from previous studies in midshipman strongly validate the value of transcriptomic analyses for linking genes to neural characters that drive behavior.

**Conclusions:**

Transcriptome analyses highlighted a suite of molecular mechanisms that regulate vocalization over behaviorally relevant timescales, spanning milliseconds to hours and seasons. To our knowledge, this is the first comprehensive characterization of gene expression in a dedicated vocal motor nucleus. Candidate genes identified here may belong to a conserved genetic toolkit for vocal motoneurons facing similar energetic and neurophysiological demands.

**Electronic supplementary material:**

The online version of this article (doi:10.1186/s12864-015-1577-2) contains supplementary material, which is available to authorized users.

## Background

Vocal-acoustic communication is a prominent feature of vertebrate social behavior. Despite having evolved diverse peripheral sonic organs, brainstem vocal networks that control vocalization are remarkably conserved across vertebrate taxa [1,2]. In tetrapods and several clades of fishes, sound production relies upon temporally stable modulations of acoustic waveforms over millisecond timescales [3-6]. Although vocal networks in several vertebrate lineages achieve high degrees of synchrony and precision (e.g., [7-10]), it is unknown whether these properties depend on a suite of conserved genetic/molecular mechanisms.

Recent microarray and genome analyses in a songbird, the zebra finch (*Taeniopygia guttata*), have advanced our understanding of the relationship between genes and vocal behavior by identifying transcription factors and gene networks activated in forebrain song nuclei [11-13], including during singing [13-15]. Similar large-scale gene expression studies in other species that aim to link gene expression to behavior typically use whole brains or regions containing multiple neuron types (e.g. [11-14,16-19]). While these studies provide significant insight into the genetic regulation of behavioral states, cellular and network level interpretations of results are confounded by the complexity of the underlying neural structure or behavior.

For the vast majority of vocal vertebrates that currently lack either genomic data or species-specific microarrays, no large-scale expression study has examined genes whose products directly support the function of a single neuronal population devoted to sound production. Here, we identify candidate molecular mechanisms contributing to the neural coding of vocal behavior in the plainfin midshipman fish (*Porichthys notatus*), an established model for the neural basis of acoustic communication. In order to link gene expression to neurophysiological events that directly translate to behavior, we focused RNA sequencing (RNA-seq) transcriptome analyses on a single hindbrain nucleus that is dedicated to the temporal patterning of a simple vocal behavior.

**Figure 1:**
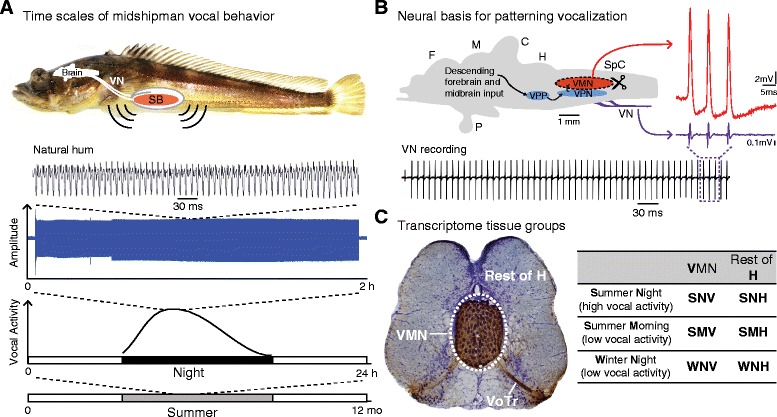
Midshipman is a neuroethological model for studying neural control of vocalization. **A)** Midshipman vocal behavior varies across a range of time scales. Picture of a midshipman fish (top) with schematic side view of the brain, vocal nerve (VN), and sonic swimbladder (SB), the sound-producing organ with vocal muscles (red) attached to the sidewalls. Continuous hums can last from mins to >1 h, as shown in the 1.85 h hum recorded from a captive male (blue trace), and are produced repetitively throughout the night during the summer breeding season (bottom). Close-up of natural hum is adapted from [25]. **B)** Schematic saggital view of the midshipman brain showing the hindbrain vocal pattern generator that consists of the vocal pre-pacemaker nucleus (VPP), vocal pacemaker nucleus (VPN), and vocal motor nucleus (VMN) (left). Forebrain and midbrain vocal centers drive the hindbrain pattern generator, which provides a precise and synchronous code that controls sonic muscle contraction in a one-to-one manner (compare the series of sound pulses in the natural hum in **A** to the vocal nerve potentials directly to the right in the bottom trace in **B**. Extreme temporal precision of motoneuron firing is shown by corresponding traces from an intracellular VMN recording (red) and VN recording (purple; adapted from [8]). Trace of a long duration VN recording (bottom) adapted from [39]. **C)** Tissue groups used for transcriptome analysis and their notations. Left: a transverse section at the level of VMN showing bilateral, transneuronal biocytin labeling in VMN and the vocal tract (VoTr) (see [27]); each VMN innervates the ipsilateral vocal SB muscle. In this study, we surgically separated the midline pair of VMN from the surrounding hindbrain tissue (Rest of H). Right: abbreviations of sample groups according to the tissue and time of collection, used throughout this paper.

Midshipman belong to an order of highly vocal teleost fish known as toadfishes (Batrachoidiformes) [20] that produce different call types depending on social context [21,22]. Nest-guarding males, which are used in this study, produce several call types, including a long duration advertisement “hum”. The hum is a stable, high frequency (100 Hz at ~16°C) call that is energetically demanding and essential for mate attraction [23]. Hums are produced continuously for minutes to hours, including in captivity (e.g., see 1.85 h hum in Figure 1A), throughout the night during the summer breeding season [22,24-26].

In midshipman, well-delineated forebrain, midbrain, and hindbrain nuclei comprise a vocal network [27-29] (Figure 1B). The collective output of this network determines the natural vocalization’s fine (e.g. fundamental frequency) and gross (e.g. duration) temporal structure [30]. This motor command is relayed to a single pair of dedicated vocal muscles by way of the hindbrain vocal motor nucleus (VMN), which forms a hindbrain vocal central pattern generator (CPG) with separate premotor pacemaker and prepacemaker nuclei (Figure 1B) [8,30-36]. Although forebrain and midbrain vocal nuclei gate and modulate hindbrain vocal CPG output and are highly sensitive to hormone action [34,37-39], the surgically isolated hindbrain vocal CPG region is both necessary and sufficient for generating the vocal command signal, and remains sensitive to hormonal modulation [34].

VMN’s intrinsic and network neuronal properties drive synchronous and stable population-level firing that faithfully amplifies premotor temporal input [8]. Each VMN spike leads to a single sound pulse, resulting in a one-to-one translation between VMN output and the temporal properties of natural vocalization, such as call duration and fundamental frequency (compare Figure 1A and B). Terrestrial vertebrates such as songbirds, bats, and primates rely on vocal networks that integrate input from breathing circuits [40-44] to drive multiple vocal and respiratory muscles. In contrast, the simpler vocal systems of fishes do not rely on airflow and engage a single pair of sonic muscles, providing a straightforward link between gene expression, neural networks, and behavior [8,10].

In addition to the direct translation between neurophysiological activity and behavior, the midshipman VMN provides several advantages for revealing molecular mechanisms underlying vocal patterning. First, the paired midline VMN together (Figure 1C) form a highly interconnected, homogenous population of ~4000 vocal motoneurons [8,45], inclusive of surrounding glial cells and presynaptic inputs that support VMN network function, which can be excised *in-toto* for focal molecular analysis of a single brain nucleus [46]. Second, a set of intrinsic (low baseline excitability, rapid membrane repolarization) and network (dense excitatory and inhibitory inputs; electrotonic coupling) neuronal properties of the VMN are well characterized [8]. These properties guide the identification of candidate genes encoding specific molecular counterparts that likely contribute to VMN’s extreme population-level synchrony on a millisecond timescale (Figure 1B). Third, midshipman vocal behavior follows predictable daily and seasonal cycles [22,25,26,39]. This allows us to utilize temporal variation to identify genes driving changes in vocal network excitability at different daily and seasonal time points [25] (Figure 1C). Fourth, a large body of work that documents hormonal modulation of the vocal system at multiple levels of analyses (neuroanatomy, neurophysiology, qPCR quantification, behavior) informs the functional significance of gene expression patterns (see [47,48]).

**Figure 2:**
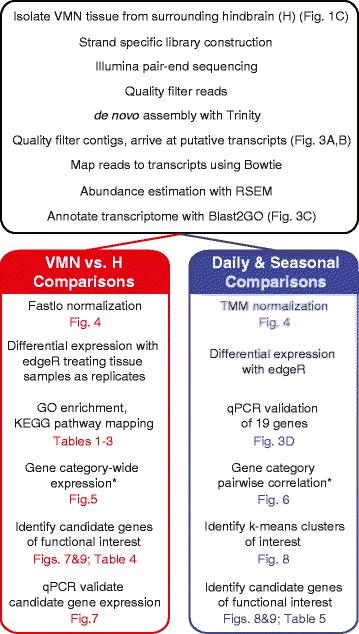
Schematic of analytic approaches with associated figure and table numbers. We took a two-pronged approach in analyzing the vast array of data generated from RNA-seq. First, for VMN vs. H comparisons, we used a cyclic loess method, fastlo, to eliminate a non-linear tissue-dependent skew between datasets (left, red). We then treated time point samples as replicates for each tissue for differential expression with edgeR. Second, we followed the Trinity-supported downstream analyses of clustering differentially expressed transcripts and focused these analyses on clusters with biologically relevant expression patterns across the day and season (right, blue). Additionally, we analyzed expression and correlation patterns of transcripts belonging to six broad gene functional categories. *Genes were grouped in broad categories regardless of whether they were differentially expressed.

We aimed to identify molecular pathways specific to VMN function by comparing patterns of gene expression in the VMN to the surrounding hindbrain (**H**) across daily and seasonal time points of high and low sound production (Figure 1A,C). As outlined in Figure 2, we focused first on between-tissue differences in gene expression to identify candidate gene pathways important for VMN function, then on within-tissue comparisons to identify candidate gene pathways with biologically relevant expression patterns across the day and season.

By harnessing the extensive knowledge of the midshipman vocal network, the VMN transcriptome elucidated a set of molecular pathways underlying neuronal excitability in a motor nucleus that instructs vocal patterning across multiple timescales, from milliseconds to hours and seasons. Our results directly inform future studies using molecular, anatomical, and neurophysiological methods to validate the function and cellular localization of candidate genes in the VMN. We propose that the candidate genes and molecular pathways identified here may belong to a shared genetic toolkit for vocal motoneurons in many species that face similar energetic and neurophysiological demands. Thus, our results will inform future comparative studies to achieve a broader understanding of the molecular machinery required for vocalization.

## Results and discussion

In order to globally characterize molecular pathways governing vocal motor patterning, we compared the transcriptome of surgically isolated VMN to H tissue at three time points corresponding to high (reproductive summer night) and low (reproductive summer morning and non-reproductive winter night) vocal activity (Figure 1C). In order to minimize activity-induced gene-expression, we used males who had not been humming prior to sacrifice. See Methods, and Figures 1 and 2 for explanations of sample groups and analysis pipelines.

### Transcriptome assembly, annotation, and qPCR validation

**Figure 3:**
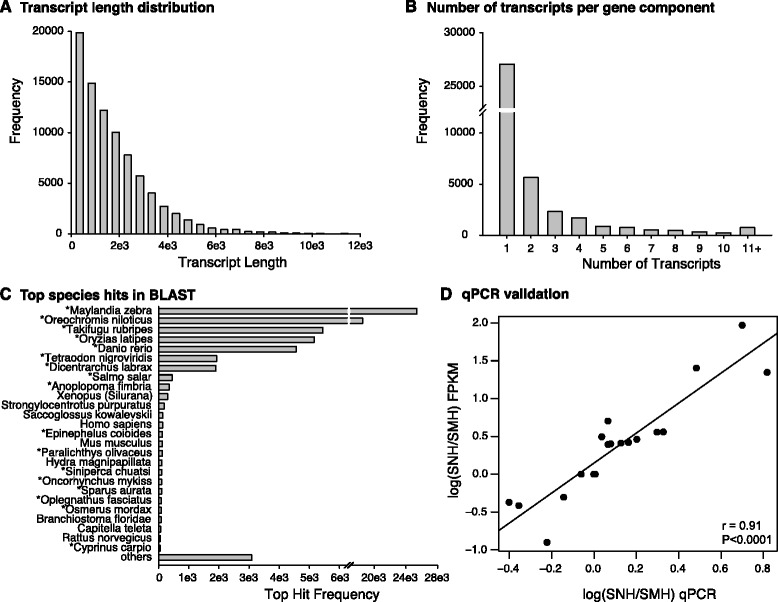
Transcriptome assembly quality assessment. **A)** Transcript length distribution. **B)** Number of transcripts per gene component as determined by Trinity. **C)** Top species hits from BLAST against NCBI’s nr database, first nine species are teleost fish sequences out of a total of 17 teleost species within the list, indicated by *. **D)** Quantitative PCR (qPCR) was used to verify transcriptome-derived FPKM values of 19 genes in SNH and SMH tissue groups. Pearson’s correlation analysis showed that qPCR and transcriptome measured ratios of SNH/SMH are significantly correlated with each other.

Sequencing using the Illumina HiSeq2000 system yielded approximately 200 million 100 bp reads across all brain groups used here and ear sample groups used for a companion study [49]. Over 90% of the raw reads survived quality filtering and trimming, resulting in 21.4 ± 2.8 million (mean ± S.D.) paired-end reads per brain sample. Our initial assembly produced 293,702 contigs, with a mean length of 1006.86 ± 1259.72 bp. After discarding contigs with open reading frames (ORFs) of less than 50 amino acids and lowly supported transcripts (isoforms) with less than 1% mapped reads for the gene (component), the final transcriptome contained 83,967 assembled transcripts, with a mean length of 1713.57 ± 1585.21 bp (range 201–18,637; N50 = 2647), representing 40,656 gene components across brain and ear sample groups. The transcript length distribution and number of transcripts per gene are shown in Figure 3A and B. The mean GC content for this filtered transcriptome was 48.22%. Of the 83,967 transcripts, 46,629 (55.5%) contained complete ORFs, comparable to a recent zebra finch transcriptome [50]. Additionally, CEGMA (Core Eukaryotic Genes Mapping Approach) analysis of conserved eukaryotic genes (CEGs), determined that our filtered transcriptome contained 453 full-length CEGs of the complete set of 458 CEGs (99%), and 238 of the more-conserved set of 248 CEGs (96%) [51,52]. Blast2GO annotation resulted in 74,000 (88.1%) of our assembled transcripts with significant annotation hits, comparable to or above reported rates (see [18,53,54]). Seventeen of the top 26 blast hit species were teleost fish, with the top nine all being teleosts (Figure 3C). There were 77,285 transcripts, representing 35,983 gene components, expressed in brain samples (>0 counts in at least one brain sample).

Using qPCR, we validated the relative abundances of 19 transcripts from two of the same, pooled H samples that were submitted for Illumina sequencing: reproductive summer morning (SMH) and reproductive summer night (SNH). Our results showed a tight and highly significant correlation between the log-normalized SNH/SMH ratios by qPCR and FPKM (fragments per kilobase of transcript per million reads mapped) (Pearson’s r = 0.91, P < 0.0001; Figure 3D). Additionally, expression levels of nine candidate genes also showed significant correlation between RNA-seq and qPCR values (see section below). Together, these results validated our transcriptome-predicted abundances and demonstrated the high quality of our *de novo* assembled transcriptome.

### VMN versus H tissue comparisons

#### Overview

**Figure 4:**
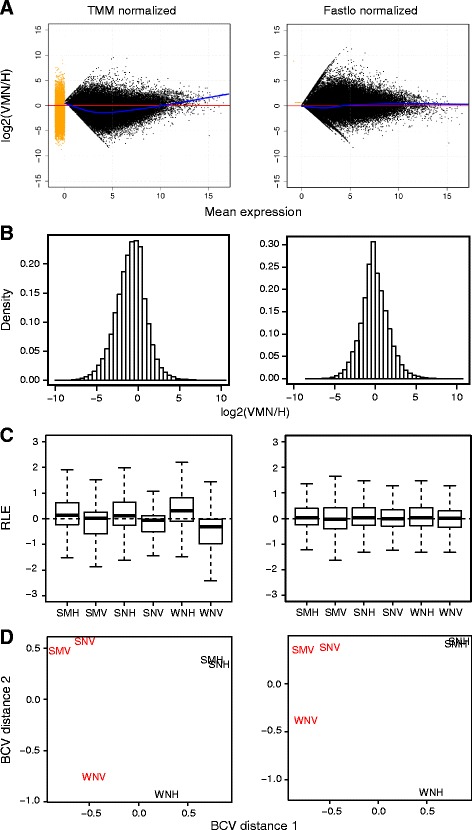
Evaluating TMM and fastlo normalization of tissue comparisons. **A)** Plots of log2(VMN/H) ratios (y axis) versus mean expression across all sample groups (x axis). N = 76,878 genes. Alignment along 0 horizontal is expected. Fitted loess lines are shown in blue. **B)** Histograms of VMN/H mean ratios. Even with peak at 0 indicates good normalization. **C)** Box plots of each sample group. Medians are expected to align along 0 with even distribution about the median across sample groups. RLE: Relative log ratio. **D)** Multidimensional scaling plots showing similarities between VMN (red) and H (black) sample groups. Samples show biologically relevant grouping by tissue and by season. BCV: biological coefficient of variation.

In order to identify molecular pathways and candidate genes important for VMN function, we first focused on between-tissue comparisons. The cyclic loess “fastlo” normalization method [55], as opposed to trimmed mean of M values (TMM) normalization [56], successfully removed the nonlinear tissue-dependent skew (Figure 4A-C), while preserving biologically relevant sample group similarities as seen in the clustering of samples by tissue and season in the multidimensional scaling plot (Figure 4D). By conservatively treating the three sample groups collected at different times as biological replicates from which to estimate transcript-wise dispersion values in edgeR’s differential expression analysis [57], we identified 1,717 transcripts significantly upregulated in H and 2,948 transcripts upregulated in VMN, which were the focus of downstream analyses.

#### GO term enrichment

**Table 1:** **Enriched GO terms of genes upregulated in VMN compared to H**

**GO category**	**GO term**	**Name**	**FDR**
Biological process	GO:0006412	Translation	2.90E-18
	GO:0006119	Oxidative phosphorylation	3.60E-18
	GO:0022900	Electron transport chain	8.20E-18
	GO:0042775	Mitochondrial ATP synthesis coupled electron transport	1.60E-17
	GO:0042773	ATP synthesis coupled electron transport	1.60E-17
	GO:0022904	Respiratory electron transport chain	2.30E-17
	GO:0045333	Cellular respiration	2.30E-17
	GO:0044283	Small molecule biosynthetic process	3.10E-15
	GO:0055114	Oxidation-reduction process	5.10E-15
	GO:0044711	Single-organism biosynthetic process	7.40E-15
Molecular function	GO:0003735	Structural constituent of ribosome	2.30E-17
	GO:0016491	Oxidoreductase activity	1.90E-15
	GO:0003954	NADH dehydrogenase activity	6.20E-10
	GO:0050136	NADH dehydrogenase (quinone) activity	6.20E-10
	GO:0008137	NADH dehydrogenase (ubiquinone) activity	6.20E-10
	GO:0015078	Hydrogen ion transmembrane transporter activity	1.70E-07
	GO:0016655	Oxidoreductase activity, acting on NAD(P)H, quinone or similar compound as acceptor	3.50E-07
	GO:0015002	Heme-copper terminal oxidase activity	3.80E-07
	GO:0016676	Oxidoreductase activity, acting on a heme group of donors, oxygen as acceptor	3.80E-07
	GO:0016675	Oxidoreductase activity, acting on a heme group of donors	3.80E-07
Cellular component	GO:0005739	Mitochondrion	8.80E-53
	GO:0044429	Mitochondrial part	1.20E-22
	GO:0044444	Cytoplasmic part	1.70E-21
	GO:0005840	Ribosome	2.10E-21
	GO:0005743	Mitochondrial inner membrane	4.40E-20
	GO:0019866	Organelle inner membrane	6.10E-19
	GO:0070469	Respiratory chain	1.50E-16
	GO:0005740	Mitochondrial envelope	1.50E-16
	GO:0030529	Ribonucleoprotein complex	2.50E-16
	GO:0005737	Cytoplasm	3.00E-16

Significantly over represented gene ontology (GO) terms found in transcripts upregulated in VMN compared to the surrounding hindbrain (H). Top 10 GO terms from each GO category (biological process, molecular function, and cellular component) are listed in order of decreasing significance by false discovery rate (FDR).

**Table 2:** **Enriched GO terms of genes upregulated in H compared to VMN**

**GO category**	**GO term**	**Name**	**FDR**
Biological process	GO:0007154	Cell communication	7.30e-15
	GO:0044700	Single organism signaling	1.60e-14
	GO:0023052	Signaling	1.60e-14
	GO:0019226	Transmission of nerve impulse	6.50e-14
	GO:0007267	Cell-cell signaling	3.50e-13
	GO:0035637	Multicellular organismal signaling	6.90e-13
	GO:0007268	Synaptic transmission	1.20e-12
	GO:0044699	Single-organism process	1.20e-11
	GO:0044763	Single-organism cellular process	7.10e-11
	GO:0050877	Neurological system process	3.60e-10
Molecular function	GO:0005515	Protein binding	3.40e-17
	GO:0015291	Secondary active transmembrane transporter activity	1.80e-09
	GO:0015293	Symporter activity	4.00e-08
	GO:0022804	Active transmembrane transporter activity	6.90e-08
	GO:0015075	Ion transmembrane transporter activity	2.70e-07
	GO:0015294	Solute:cation symporter activity	4.00e-07
	GO:0015081	Sodium ion transmembrane transporter activity	1.20e-06
	GO:0022857	Transmembrane transporter activity	1.40e-06
	GO:0022891	Substrate-specific transmembrane transporter activity	1.70e-06
	GO:0008324	Cation transmembrane transporter activity	4.40e-06
Cellular component	GO:0031224	Intrinsic to membrane	8.00e-10
	GO:0016021	Integral to membrane	5.20e-09
	GO:0044425	Membrane part	6.70e-09
	GO:0016020	Membrane	2.40e-07
	GO:0005886	Plasma membrane	1.70e-06
	GO:0030054	Cell junction	8.30e-06
	GO:0071944	Cell periphery	1.00e-05
	GO:0045202	Synapse	1.60e-04
	GO:0097060	Synaptic membrane	3.10e-04
	GO:0044456	Synapse part	3.30e-04

Significantly over represented gene ontology (GO) terms found in sequences upregulated in the surrounding hindbrain (H) compared to the VMN. Top 10 GO terms from each GO category (biological process, molecular function, and cellular component) are listed in order of decreasing significance by false discovery rate (FDR).

We performed Fisher’s exact test on GO terms to identify gene functions that are enriched in VMN or H. By comparing the subset of genes upregulated in VMN to the entire transcriptome, we found 279 over-represented GO terms (complete list in Additional file 1: Table S1). The enrichment results overwhelmingly highlighted the importance of ATP production in VMN, with 29% of the enriched GO terms relating to aerobic metabolism (Additional file 1: Table S1). This high metabolic activity in the VMN is exemplified by the 10 most significantly enriched GO terms (Table 1). Although few VMN enriched GO terms were related to neural transmission functions, the GO terms “neurotransmitter biosynthetic process” and “choline O-acetyltransferase activity” were enriched, consistent with VMN motoneurons being cholinergic [58] (Additional file 1: Table S1). Furthermore, we found several enriched GO terms related to steroid hormone signaling, including “gonadotropin secretion”, “endocrine hormone secretion”, and “sterol biosynthetic process”, indicating that the VMN is hormonally active (Additional file 1: Table S1). Finally, GO terms related to post-transcriptional processes were also enriched for VMN, including “translation” (Biological process, Table 1), “ribosome” (Cellular component, Table 1), “gene expression” (Additional file 1: Table S1), and “RNA processing” (Additional file 1: Table S1), indicating that VMN is translationally active.

In contrast, 75% of the 130 GO terms enriched in transcripts upregulated in H were related to synaptic function and neurotransmission (see Additional file 1: Table S2 for complete list). The top 10 *Biological Process* GO terms were all related to cell signaling and synaptic transmission, the top 10 *Molecular Function* terms were related to membrane transporter activity, and the top 10 *Cellular component* GO terms were related to the synapse or membrane (Table 2). The observation that H contains a higher diversity of neurotransmission genes is consistent with H containing diverse motor and non-motor nuclei as well as abundant white matter (see [27]) compared to the high homogeneity of motoneurons in VMN. In contrast to VMN, we found no enriched terms related to steroid hormones in H, although “neuropeptide signaling pathway” was enriched (Additional file 1: Table S2).

#### KEGG (Kyoto Encyclopedia of Genes and Genomes) pathways

**Table 3:** **KEGG pathways mapped to transcripts differentially upregulated in VMN vs. H**

**VMN**		**H**	
**Pathway**	**# seqs**	**Pathway**	**# seqs**
Purine metabolism	60	Phosphatidylinositol signaling system	19
Pyrimidine metabolism	29	Glycerophospholipid metabolism	15
Oxidative phosphorylation	28	Purine metabolism	12
Aminoacyl-tRNA biosynthesis	25	Lysine degradation	12
Carbon fixation pathways in prokaryotes	16	Glycerolipid metabolism	11
Pyruvate metabolism	16	T cell receptor signaling pathway	9
Valine, leucine and isoleucine degradation	14	Inositol phosphate metabolism	7
Citrate cycle (TCA cycle)	13	Alanine, aspartate and glutamate metabolism	4
Porphyrin and chlorophyll metabolism	13	Arginine and proline metabolism	3
Glycolysis/Gluconeogenesis	13	Fatty acid biosynthesis	3
Glutathione metabolism	11	Glutathione metabolism	0
Steroid hormone biosynthesis	7	Steroid hormone biosynthesis	0
Steroid biosynthesis	5	Steroid biosynthesis	0
Steroid degradation	3	Steroid degradation	0

Top 10 KEGG (Kyoto Encyclopedia of Genes and Genomes) pathways with the highest number of mapped transcripts that were upregulated in VMN or H. Additionally, we show that more transcripts upregulated in VMN are mapped to glutathione methabolism, steroid and steroid hormone biosynthesis pathways.

In order to compare the relative representation of biochemical signaling pathways in VMN vs. H, we mapped differentially expressed transcripts to KEGG pathways [59] (complete list in Additional file 1: Table S3). Transcripts upregulated in VMN mapped predominantly to pathways directly related to ATP production, including “oxidative phosphorylation”, “pyruvate metabolism”, “citrate cycle (TCA cycle)”, and “glycolysis/gluconeogenesis”, which is consistent with GO term enrichment results that also indicated high levels of metabolic activity in VMN. Pathways unique to transcripts upregulated in VMN also included those involved in antioxidant defense against oxidative stress, such as “glutathione metabolism” [60], indicating that VMN is capable of combating reactive oxygen species generated by aerobic metabolism. Additionally, we found steroid hormone related pathways (“steroid hormone biosynthesis”, “steroid biosynthesis”, “steroid degradation”; Table 3, Additional file 1: Table S3) that support GO term enrichment results highlighting VMN as a hormonally active nucleus. Transcripts in the “carbon fixation pathways in prokaryotes” found in VMN were confirmed by BLAST to have high identity to transcripts of other fish and likely represent metabolic genes conserved from bacteria through vertebrates. In contrast, transcripts upregulated in H mapped predominantly to phospholipid metabolism and signaling pathways (Table 3). Phospholipids are important components of cell membranes and precursors to important secondary messengers such as diacyl glycerol and inositol 1,4,5-triphosphate [61].

Together, KEGG pathway and GO term enrichment results showed that VMN is metabolically, hormonally, and post-transcriptionally active. In contrast, transcription in the surrounding hindbrain is devoted to synaptic transmission and membrane processes. It has been estimated that acoustic courtship in ectotherms requires an eight-fold increase from resting metabolic rate [62,63]. Results highlighting the importance of cellular respiration in VMN are consistent with its ability to sustain high frequency firing that drives advertisement hums up to hours (Figure 1A) repetitively throughout a single night of courtship-related activity [22,24,26]. While studies have extensively characterized the high aerobic demands of “superfast” muscles in midshipman and closely related toadfish, among the fastest contracting skeletal muscles in vertebrates [64,65], our results indicate a concomitantly high metabolic demand in the motoneurons that drive these muscles.

#### Gene category analyses

**Figure 5:**
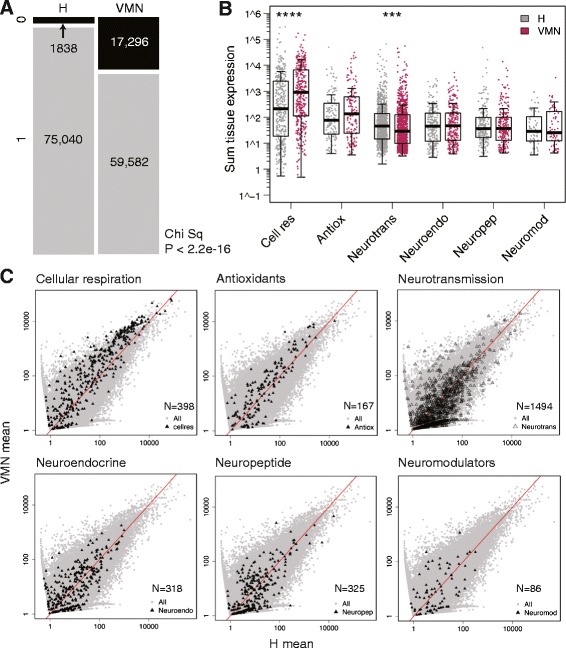
Tissue comparison of functional category expression. **A)** Raw counts from the brain transcriptome were dichotomized into expressed (1, gray) or not expressed (0, black) based on the summed tissue values. Compared to VMN, H has significantly more expressed genes. Chi sq: Chi squared test. **B)** Normalized abundances for each unique transcript expressed were summed from VMN or H sample groups, box-cox transformed, and subjected to one-way ANOVA for tissue comparison, with the Bonferroni corrected alpha level set at 0.008. Cellular respiration transcripts had significantly higher expression in the VMN while neurotransmission transcripts were higher in H. ****P < 0.0001, ***P = 0.0005. **C)** Mean VMN vs. H expression levels (fastlo-normalized) of transcripts within each of the six gene categories (black triangles) are plotted on top of all transcripts (gray dots). N: number of transcripts in each category. Cell res: cellular respiration; Antiox: antioxidants; Neurotrans: neurotransmission; Neuroendo: neuroendocrine; Neuropep: neuropeptide; Neuromod: neuromodulators.

**Figure 6:**
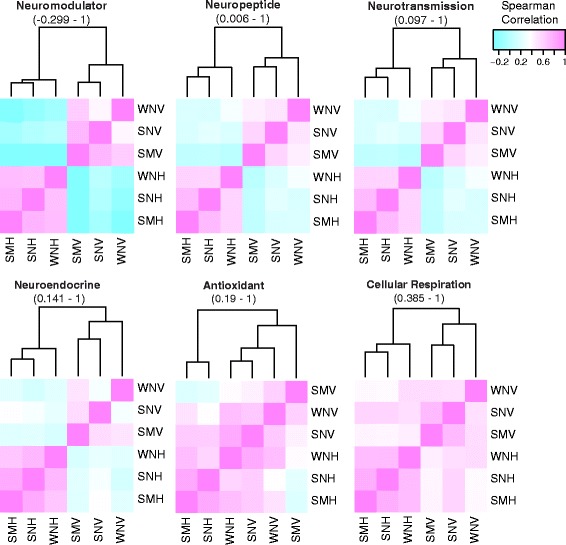
Spearman correlation heatmaps of functional gene categories. Heatmaps are generated from Spearman correlation coefficients (rho) for each pair of samples based on TMM-normalized FPKM values. Values in parenthesis are the ranges of rho.

Guided by the above results and previous studies of VMN characteristics, we wanted to compare expression levels of transcripts belonging to different functional categories with significant implications for cellular and network level excitability. Thus, regardless of differential expression, we selectively analyzed transcripts belonging to the following functional categories: neurotransmission (ion channels and neurotransmitter receptors), neuroendocrine (genes related to steroid and thyroid hormone function), neuropeptides, neuromodulators, cellular respiration, and antioxidants (Figures 5 and 6). Transcripts for each category are listed in Additional file 1: Table S4.

While the overall pool of expressed transcripts was smaller in VMN than in H (Chi squared test, P < 0.0001) (Figure 5A), transcript abundances were comparable between tissues in most gene categories (Figure 5B). Two exceptions were cellular respiration, which had significantly higher expression in VMN (ANOVA, F = 30.84, P < 0.0001), and neurotransmission, which had significantly higher expression in H (ANOVA, F = 12.07, P = 0.0005) (Figure 5B,C), substantiating our interpretations of GO term enrichment results as discussed above (Tables 1–2).

Pairwise Spearman correlation analyses revealed that overall, cellular respiration had the highest correlation between sample groups (Figure 6). Because the Spearman coefficient is derived from calculating the Pearson correlation of transcript abundance ranks between two samples, these results demonstrate that although cellular respiration expression levels were higher in VMN (Figure 5), the relative abundances of transcripts to one another is conserved across sample groups (Figure 6). In contrast, the other functional categories, especially neuromodulators, showed lower correlation between VMN and H (Figure 6).

#### Candidate genes

**Figure 7:**
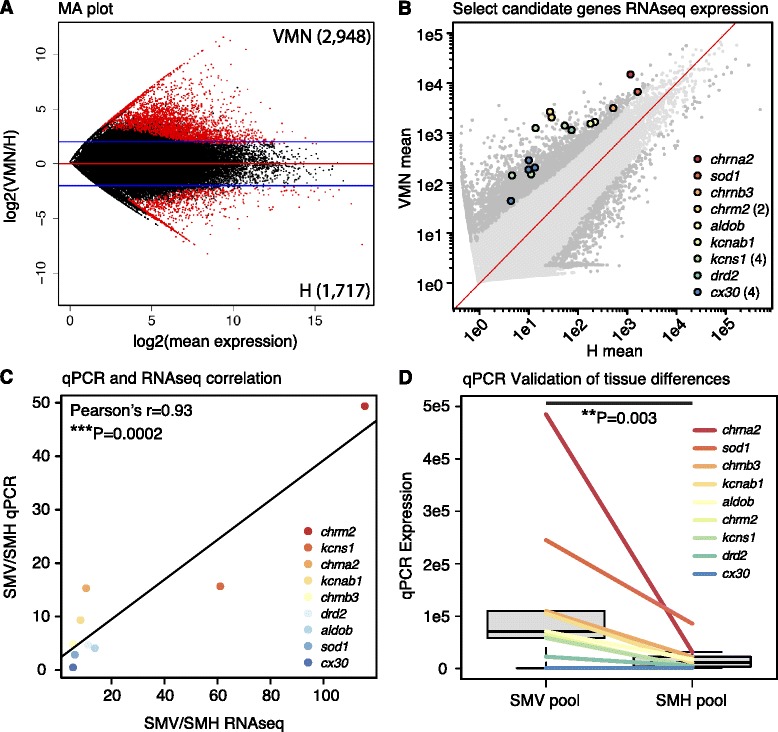
Candidate functional genes from tissue comparisons validated by qPCR. **A)** MA plot showing log2 VMN/H mean ratios by average expression level for each transcript. Mean values were calculated from the three sample groups for each tissue. Transcripts with positive log ratios are higher in VMN, while transcripts with negative log ratios are higher in H. The numbers of significantly upregulated transcripts for each tissue are shown in parentheses. Red dots indicate significantly differentially expressed transcripts in VMN or H. Blue lines indicate log2 values of +/− 2, representing fold-change of 4. **B)** Nine candidate genes that were chosen for qPCR verification are plotted on top of a scatter plot of VMN mean vs. H mean values for all transcripts, calculated from the three sample groups for each tissue. Number of differentially expressed isoforms per gene is indicated in parentheses. Dark gray dots are significantly differentially expressed transcripts in VMN or H. The line of unity is in red. **C)** Correlation of SMV/SMH ratios derived from qPCR data and fastlo-normalized RNAseq data. For RNAseq data, ratios were calculated from the average expression of all isoforms within a gene component. Linear regression line is in black. **D)** qPCR validation of SMV and SMH expression for the candidate genes showing significant upregulation of candidate genes in SMV.

**Table 4:** **Functionally important candidate genes upregulated in VMN**

**Category**	**Blast2GO description**
Neurotransmission	Anoctamin-5-like isoform x4
	ATP-sensitive inward rectifier potassium channel 8-like
	Calcium-activated potassium channel subunit alpha-1-like (*kcnma1*) isoform 3
	Gamma-aminobutyric acid receptor subunit alpha-3-like (*gabra3*)
	Gamma-aminobutyric acid receptor subunit alpha-5-like (*gabra5*)
	Gamma-aminobutyric acid receptor subunit pi-like (*gabrp*)
	Gamma-aminobutyric acid type b receptor subunit 1-like
	Gap junction beta-6 (*cx30*)
	Glutamate ionotropic kainate 3-like
	Glutamate ionotropic kainate 5-like
	Glutamate receptor delta-1 subunit-like
	Glycine receptor subunit beta
	Glycine receptor subunit beta-like
	Kv channel-interacting protein 4-like
	Muscarinic acetylcholine receptor m2 (*chrm2*)
	Neuronal acetylcholine receptor subunit alpha-2-like (*chrna2*)
	Neuronal acetylcholine receptor subunit alpha-3-like
	Neuronal acetylcholine receptor subunit alpha-9-ii-like
	Neuronal acetylcholine receptor subunit beta-2-like
	Neuronal acetylcholine receptor subunit non-alpha-3-like (*chrnb3*)
	Potassium sodium hyperpolarization-activated cyclic nucleotide-gated channel 2-like
	Potassium voltage-gated channel subfamily b member 2 (*kcnb2*/Kv2.2)
	Potassium voltage-gated channel subfamily c member 3-like (*kcnc3*/Kv3.3) isoform x1
	Potassium voltage-gated channel subfamily e member 1-like
	Potassium voltage-gated channel subfamily h member 5
	Potassium voltage-gated channel subfamily kqt member 2-like (*kcnq2*/Kv7.2)
	Potassium voltage-gated channel subfamily s member 1-like (*kcns1*/Kv9.1)
	Potassium voltage-gated channel subfamily s member 3-like
	Sodium channel protein type 5 subunit alpha
	Sodium channel subunit beta-4-like
	Transient receptor potential cation channel subfamily m member 2-like
	Two pore calcium channel protein 1-like
	Voltage-dependent l-type calcium channel subunit alpha-1f-like
	Voltage-dependent r-type calcium channel subunit alpha-1e
	Voltage-gated potassium channel subunit beta-1 isoform 1 (*kcnab1*)
	Voltage-gated potassium channel subunit beta-2-like isoform 1
Steroid pathway	3-keto-steroid reductase-like (*hsd17b7*)
	Androgen receptor alpha (*ar-a*)
	Androgen-induced gene 1 protein
	Dihydroxyvitamin d 24- mitochondrial-like isoform 1
	Estrogen receptor beta 2 (*esr2*)
	Estrogen-related receptor alpha
	Hydroxysteroid 11-beta-dehydrogenase 1-like (*hsd11b1l*)
	Lanosterol 14-alpha demethylase-like
	Ovarian aromatase (*cyp19a1a*)
	Sterol regulatory element-binding protein 1
	Sterol regulatory element-binding protein 2
Neuropeptides/peptide hormones	Atrial natriuretic peptide-converting enzyme-like
	Calcitonin gene-related peptide precursor
	Cholecystokinin type a receptor
	Growth hormone receptor
	Inhibin beta b chain-like
	Insulin gene enhancer protein isl-1
	Insulin-like growth factor binding protein 1
	Insulin-like growth factor i
	Neuropeptide b precursor
	Peptide yy-like
	Pituitary adenylate cyclase-activating polypeptide type i receptor-like
	Vasoactive intestinal polypeptide receptor 1-like
Thyroid hormone	Thyroid hormone receptor alpha
	Thyroid hormone receptor-associated protein 3-like
	Thyroid receptor-interacting protein 6-like
Other neuromodulators	Adenosine receptor a1-like
	D-like dopamine receptor-like (*drd2*)
	Melatonin receptor type 1a-like
	Prostaglandin e synthase 2-like
	Prostaglandin f2-alpha receptor-like
Antioxidants	Cu/Zn superoxide dismutase (*sod1*)
	Glutaredoxin 3
	Glutaredoxin-related protein 5
	Glutathione peroxidase 4b (*gpx4b*)
	Glutathione s-transferase (*gst*)
	Peroxiredoxin 6
	Thioredoxin domain-containing protein 17
	Thioredoxin-dependent peroxide mitochondrial precursor
	Thioredoxin-like protein 1
	Thioredoxin-like protein 4a

Gene descriptions with neurotransmission, neuroendocrine, and antioxidant functions from transcripts upregulated in VMN compared to surrounding hindbrain. Genes that are mentioned in the text or shown in figures have gene symbols in parentheses.

Supporting the hypothesis that VMN expresses a unique molecular toolkit dedicated to vocal motor coding, the majority of functionally important transcripts upregulated in VMN (Table 4) were unique from those upregulated in H (Additional file 1: Table S5). Among the transcripts significantly upregulated in VMN relative to H (Figure 7A), we identified those with neurotransmission functions, including ligand- and voltage-gated ion channels as well as metabotropic neurotransmitter receptors (Table 4). We also identified transcripts with neuroendocrine functions, including steroid signaling and biosynthesis, neuropeptides and peptide hormones, and other neuromodulators (Table 4). Neurotransmission and neuroendocrine functions were the focus because of relevance to neural excitability and previous midshipman studies focusing on these mechanisms.

#### Candidate neurotransmission genes

VMN’s synchronous and temporally precise activity is dependent on both synaptic interactions of the network as well as intrinsic properties of individual motoneurons [8]. There were 57 transcripts with 34 unique neurotransmission gene annotations upregulated in the VMN (Table 4). These candidate genes included those supporting two of VMN’s major network components: electrical synapses formed by gap junctions and GABAergic inhibition [8], both of which are also prominent mechanisms promoting network-level synchrony in other systems [66,67]. The presence of elevated gap junction subunit expression in VMN (Table 4) affirmed previous evidence of electrotonic coupling in VMN, demonstrated by electron microscopy [68], transneuronal dye diffusion [8,27], and intracellular neurophysiology [8]. The multiple GABA receptor subunits upregulated in VMN (Table 4) likely provide the prominent network-dependent afterhyperpolarization observed in VMN motoneurons, supported by the pharmacological blocking of GABA_A_ receptors that severely reduced the duration and amplitude of VMN output [8].

VMN’s intrinsic neuronal properties are likely mediated by several voltage-gated potassium channel (KCN/Kv) subunits that have known functions in controlling neuronal excitability (Table 4; we report potassium channel gene names by both the KCN and Kv nomenclature systems [69,70]). First, VMN showed increased expression of *potassium voltage-gated channel subfamily c member 3-like* (*kcnc3*/Kv3.3) subunits (Table 4), which contributes significantly to action potential repolarization and recovery of sodium channels from inactivation (e.g. [71]), permitting high-frequency repetitive firing that is a key feature of VMN motoneurons [8]. Second, it has been shown that while nonfunctional alone, KCNS1/Kv9.1 subunits decrease KCNB1/Kv2.1 currents when coassembled in heteromeric channels, which was modeled to increase the firing fidelity of a simulated neuron to a 100 Hz stimulus [72]. The potential interaction of our candidate *potassium voltage-gated channel subfamily s member 1-like* (*kcns1*/Kv9.1) and *potassium voltage-gated channel subfamily b member 2* (*kcnb2*/Kv2.*2*) subunits (Table 4) may therefore regulate VMN firing frequency that directly determines the pulse repetition rate and fundamental frequency of natural vocalizations (see [32]). KCNS1/Kv9.1 subunits also decrease the conductance of KCNC/Kv3 subunits, mentioned above, when they form heteromeric channels [73]. Third, channels composed of *potassium voltage-gated channel subfamily kqt member 2-like* (*kcnq2*/Kv7.2) subunits, also elevated in VMN (Table 4), dampen neural excitability by being partially activated at resting membrane potentials [74]. We propose *kcnq2*/Kv7.2 underlies the low baseline excitability of VMN neurons, which contributes to their ability to fire with high fidelity to presynaptic input [8] from the vocal pacemaker nucleus that sets call frequency [30]. In the fully aquatic frog *Xenopus laevis*, male laryngeal motoneurons that drive vocalizations also have a strong low-threshold potassium current [10], leading to the hypothesis that low intrinsic excitability conferred by high *kcnq2*/Kv7.2 expression may be a shared feature of vertebrate vocal motoneurons.

Although we identified transcripts of candidate neurotransmission genes predicted *a priori* to be important for VMN function, we also found candidates previously not well studied in this system. One example is evidence of cholinergic input to VMN, supported by choline acetyltransferase staining of somata in the vocal premotor pacemaker region of midshipman ([58], A. Bass, unpub observ), that has potentially significant contributions to VMN function. The alpha and beta subunits of neuronal nicotinic receptors found in our candidate list (Table 4) could mediate fast, postsynaptic action of acetylcholine [75], or modulation of presynaptic neurotransmitter release, including GABA [76], shown to play a prominent role in regulating VMN output (see above). Furthermore, evidence from other motor systems indicates cholinergic control of motoneuron excitability and firing frequency [77-79], including actions mediated via postsynaptic muscarinic receptors and KCNB1/Kv2.1 potassium channels [77,78,80,81], transcripts of which were elevated in VMN: *muscarinic acetylcholine receptor m2* (*chrm2*) and *kcnb2*/Kv2.2 (Table 4). Another target of muscarinic modulation is the KCNQ/Kv7 family of voltage gated potassium channels that decrease baseline neural excitability, of which *kcnq2*/Kv7.2 is found among our candidates (discussed above; Table 4) [74].

Importantly, many of the candidate ion channels we note above are ideal targets for pharmacological validation using selective blockers (e.g. see [82] for *kcnq2*/Kv7.2 blockers, [71] for *kcnc*/Kv3 blockers, [69] for other potassium channel blockers; [83] for muscarinic and [84,85] for neuronal nicotinic acetylcholine receptor blockers; [86] for *gabra5* blocker). It is also worth highlighting that both *kcns1*/Kv9.1 and *kcnq2*/Kv7.2 mRNA levels were elevated in four major telencephalic song control nuclei in the zebra finch [12], with *kcns1*/Kv9.1 showing additional label in the tracheosyringeal division of the hypoglossal motor nucleus (nXIIts) (see the ZEBrA database, Oregon Health & Science University, Portland, OR 97239; http://www.zebrafinchatlas.org), the brainstem region representing VMN’s analogue, if not homologue (see [3]).

#### Candidate neuroendocrine and neuromodulator genes

Both behavioral and neurophysiological studies have shown that steroids exert rapid and robust effects on vocal behavior and the output of the vocal CPG via classical steroid receptors in midshipman and a closely related toadfish species (e.g. [34,87]). Consistent with these studies, steroid receptors *androgen receptor alpha* (*ar-a*) and *estrogen receptor beta 2* (*esr2*) were both upregulated in the VMN (Table 4). The estradiol synthetic enzyme *ovarian aromatase* (*cyp19a1a*) was upregulated in the VMN (Table 4), consistent with previous studies showing dense aromatase protein and mRNA expression in a dorsal layer of glial cells that surrounds and projects throughout the VMN [88]. The steroidogenic enzyme *3-keto-steroid reductase-like* (*hsd17b7*)*,* responsible for the interconversion between estrone and estradiol [89], was also elevated in the VMN (Table 4). Additionally, we found higher VMN expression of *hydroxysteroid 11-beta-dehydrogenase 1-like* (*hsd11b1l*), a crucial enzyme that converts 11-beta-hydroxytestosterone to 11-ketotestosterone, and cortisol to the inactive metabolite cortisone [90] (Table 4). 11-ketotestosterone and cortisol are the major circulating androgen and glucocorticoid, respectively, in type I male midshipman that are the source of tissues used here (see [91]), and cause a rapid increase in vocal CPG output duration within five minutes of systemic injection [33,34]. Thus, many of the candidate neuroendocrine transcripts upregulated in VMN have direct relevance to VMN function as corroborated by prior studies.

Additionally, we found previously under-studied neuroendocrine or neuromodulatory signaling pathways in VMN, including growth hormone, thyroid hormone, prostaglandin and dopamine, the latter of which is consistent with dense catecholaminergic input to VMN [92] (Table 4). One intriguing example is *Prostaglandin f2-alpha receptor-like*, because Prostaglandin f2-alpha is a potent sex pheromone that induces spawning behavior in fish [93], though little is known about its effects on vocal behavior.

Together, the steroid signaling candidate genes indicate that VMN exhibits high androgen and estrogen sensitivity by expressing steroid receptors and steroidogenic enzymes, consistent with prior studies showing their anatomical localization to VMN and influence on vocal CPG output (e.g. [46,94]). VMN function is also likely modulated by candidate gene products belonging to novel neuroendocrine and neuromodulatory pathways identified here.

#### *Candidate antioxidant genes*

Based on the enrichment of cellular respiration genes being upregulated in VMN, expected to generate harmful reactive oxygen species [95], we hypothesized that the VMN combats oxidative stress by expressing high levels of antioxidant enzymes and proteins. Supporting this hypothesis, we found the increased expression of several antioxidant genes in the VMN compared to H (Table 4). Notably, these included the well-studied *Cu/Zn superoxide dismutase (sod1)* enzyme (Figure 7B, C; Table 4), which catalyzes the conversion of two superoxide radicals into hydrogen peroxide [95]. The importance of *sod1* in motoneuronal function is demonstrated by human *sod1* mutations that lead to the motoneuron degenerative disease amyotrophic lateral sclerosis (ALS) [96,97]. The suite of antioxidants upregulated in VMN also included enzymes that produce or utilize glutathione for reducing reactive oxygen species, such as *glutathione s-transferase* (*gst*), which synthesizes glutathione [98], and *glutathione peroxidase 4b* (*gpx4b*), which detoxifies hydrogen peroxide [95] (Table 4). These results support our hypothesis that the VMN must combat oxidative stress incurred by high rates of cellular respiration resulting from prolonged activity [22,24,26]. Because the fish used in this study were not actively engaged in vocal activity at the time of sacrifice (see Methods), these results highlight a constitutive feature of the VMN. Based on the implication of antioxidant enzymes and proteins in motoneuron and neural degenerative diseases [96], we believe the VMN is a motor nucleus capable of withstanding extreme oxidative stress and therefore a useful model for studying the relationship between antioxidants and neural function.

#### Candidate gene qPCR validation

We chose nine candidate genes upregulated in VMN to validate expression levels with qPCR (Figure 7C). We used the same, pooled SMV and SMH RNA for generating our RNA-seq libraries to validate our findings of upregulated transcripts in the VMN. There was a high correlation of expression levels of all nine candidate genes measured by the two methods (SMV: Pearson’s r = 0.97, P < 0.0001; SMH: Pearson’s r = 0.89, P = 0.0002) (Additional file 2). Similarly, SMV/SMH fold changes calculated from qPCR correlated significantly with RNA-Seq derived fold changes (Pearson’s r = 0.93, P = 0.0002) (Figure 7C). Finally, in concordance with RNA-seq results, qPCR expression levels of candidate genes were significantly higher in SMV when compared to SMH (t = 4.22, P = 0.003) (Figure 7D). The only gene not showing the expected pattern, *cx30*, had 20 assembled isoforms and had the lowest expression among the tested candidates (Figure 7D). Altogether, qPCR results largely supported RNA-seq results.

### Seasonal and daily variation in the VMN

#### Overview

**Figure 8:**
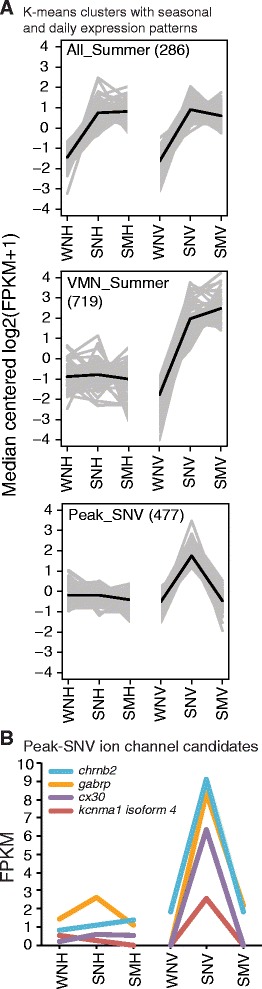
K-means clusters showing seasonal and daily patterns of gene expression. **A)** Representative K-means clusters showing upregulation in all summer sample groups, VMN summer groups, and peak expression in summer night VMN (SNV; see Figure 1C for other abbreviations). Total transcript numbers for each K-means cluster expression pattern are shown next to the cluster type names used in the text. **B)** FPKM expression levels are plotted for our candidate ion channels exhibiting peak expression in SNV. *chrnb2*: neuronal acetylcholine receptor subunit beta-2; *gabrp*: gamma-aminobutyric acid receptor subunit pi; *cx30*: connexin 30/gap junction beta-6; *kcnma1* (or Kca1.1): calcium-activated potassium channel subunit alpha-1.

We aimed to identify functionally important candidate genes whose expression levels change on a daily or seasonal basis. Hierarchical clustering of differentially expressed transcripts revealed that sample groups were most divergent across tissues (H vs. VMN samples) and reproductive state (reproductive summer vs. non-reproductive winter) (Additional file 3), consistent with the above results (Figure 4D). Furthermore, the Spearman correlation matrix showed higher within-tissue differences in the VMN than in the H (Additional file 3). Significantly differentially expressed transcripts were separated into 109 K-means clusters, from which we identified clusters showing seasonal and daily expression patterns (Figure 8A) with upregulated expression in: all summer reproductive samples (“All-Summer”: SMV, SNV, SMH and SNH), summer VMN samples (“VMN-Summer”: SMV and SNV), or SNV (“Peak-SNV”).

#### Candidate genes

**Table 5:** **Functionally important candidate genes showing daily and seasonal regulation**

**Category**	**Cluster pattern**	**Blast2GO description**
Neurotransmission	Peak-SNV	Anoctamin-10-like isoform x1
	Peak-SNV	Anoctamin-10-like isoform x4
	Peak-SNV	ATP-sensitive inward rectifier potassium channel 8-like
	Peak-SNV	Calcium-activated potassium channel subunit alpha-1-like (*kcnma1*/Kca1.1) isoform x1
	Peak-SNV	Calcium-activated potassium channel subunit alpha-1-like (*kcnma1*/Kca1.1) isoform x4
	Peak-SNV	GABA receptor subunit pi-like (*gabrp*)
	All-Summer	Gap junction alpha 1 (*cx43*)
	VMN-Summer, Peak-SNV	Gap junction beta 6 (*cx30*)
	VMN-Summer	Glutamate ionotropic kainate 4
	All-Summer	Glycine receptor subunit beta
	VMN-Summer	Glycine receptor subunit beta-like
	VMN-Summer, Peak-SNV	Neuronal acetylcholine receptor subunit alpha-3-like
	Peak-SNV	Neuronal acetylcholine receptor subunit beta-2-like (*chrnb2*)
	All-Summer	Potassium voltage-gated channel subfamily c member 4-like (*kcnc4*/Kv3.4)
	VMN-Summer	Potassium voltage-gated channel subfamily h member 5-like
	VMN-Summer	Potassium voltage-gated channel subfamily kqt member 2-like (*kcnq2*/Kv7.2)
	All-Summer	Sodium channel protein type 8 subunit alpha-like
	VMN-Summer	Sodium channel subunit beta-4-like
	VMN-Summer	Transient receptor potential cation channel subfamily m member 7-like (*trpm7*)
	VMN-Summer	Two pore calcium channel protein 1-like
Steroid receptors and metabolic enzymes	VMN-Summer	3-keto-steroid reductase-like
	All-Summer	Androgen receptor alpha (*ar-a*)
	VMN-Summer	Androgen-induced gene 1
	All-Summer	Cholesterol 25-hydroxylase-like protein member 1-like
	Peak-SNV	Estrogen receptor alpha (*esr1*)
	VMN-Summer	Glucocorticoid receptor (*nr3c1*)
	VMN-Summer	Hydroxysteroid dehydrogenase-like protein 2
	VMN-Summer	Neutral cholesterol ester hydrolase 1-like
	VMN-Summer	Oxysterol-binding protein 10 isoform 1
	VMN-Summer	Oxysterol-binding protein 8-like
	Peak-SNV	Oxysterol-binding protein 9-like isoform 3
	Peak-SNV	Sterol 26- mitochondrial-like
Peptide hormones and receptors	VMN-Summer, All-VMN	Calcitonin gene-related peptide precursor
	Peak-SNV	Growth hormone receptor
	All-Summer	Growth hormone-regulated tbc protein 1-a-like
	VMN-Summer	Insulin gene enhancer protein isl-2a-like
	All-Summer	Insulin receptor-like
	VMN-Summer	Insulin-like growth factor-binding protein 1-like
	VMN-Summer	Opioid growth factor receptor-like
	VMN-Summer	Parathyroid hormone/parathyroid hormone-related peptide receptor-like
Melatonin	All-Summer	Acetylserotonin o-methyltransferase-like

Functionally important candidate genes found in All-Summer, VMN-summer, and Peak-SNV clusters. Different transcripts with the same sequence description found in both VMN and H clusters are not listed. Additionally, duplicate descriptions from multiple isoforms within each cluster type were reported only once. Genes mentioned in the text or shown in figures have gene symbols in parentheses.

We found evidence for daily and seasonal regulation of candidate genes for specific ion channel subtypes as well as steroidogenic enzymes and receptors belonging to major hormone signaling pathways previously implicated in regulating seasonal and daily cycles of vocal motor excitability and behavior in midshipman.

#### Seasonal comparisons

**Figure 9:**
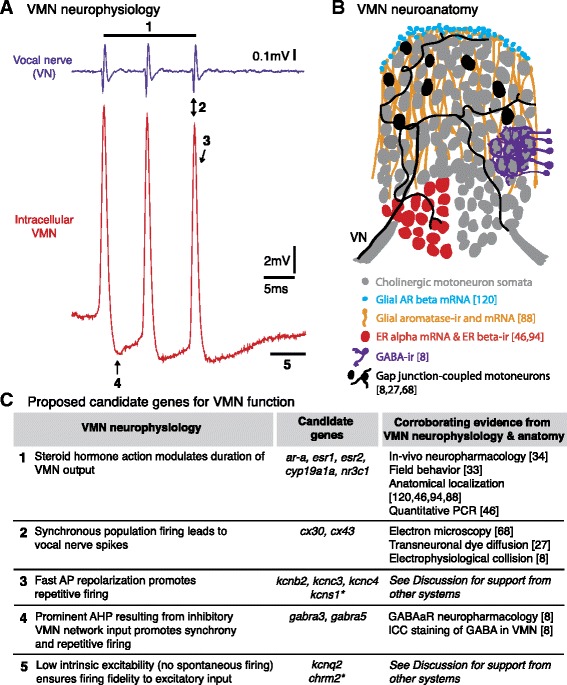
Summary of proposed candidate genes as molecular basis of known VMN properties. **A)** Known VMN neurophysiological properties. Extreme temporal precision of motoneuron firing is shown by corresponding traces from an intracellular recording (bottom, red) and VN recording (top, purple) (adapted from [8]). Numbers 1–5 correspond to intrinsic neuronal and network VMN properties listed in **C**. **B)** Schematic of known VMN neuroanatomical properties. Most of the motoneuron somata appear gray with subsets of red and black somata to highlight properties that are representative of the entire VMN. Glial expression of aromatase [88] and androgen receptor beta [120] is depicted in blue and orange. One of the black somata in the left VMN also depicts each motoneuron’s dendritic arbor that branches throughout each of the midline pair of motor nuclei and a single unbranched axon that exits via the ipsilateral vocal nerve (VN) (see [8]). The subsection of dense GABAergic innervation by cells lying outside of the VMN is also representative of the entire VMN. Abbreviations: AR: androgen receptor; ER: estrogen receptor; GABA: gamma-aminobutyric acid. **C)** For the suite of VMN properties, we identified corresponding transcripts that were significantly upregulated in the VMN compared to the surrounding hindbrain, and provide substantiating evidence from previous midshipman studies (see reference list for numbered citations). Abbreviations: *ar-a*: androgen receptor alpha; *esr*: estrogen receptor; *cyp19a1a*: aromatase; *nrc3c1*: glucocorticoid receptor; *cx*: connexin (gap junction); AP: action potential; *kcn*: voltage-gated potassium channels; AHP; afterhyperpolarization; *gabra*/GABAaR: GABA_A_ receptor; ICC: immunocytochemistry; *chrm2*: muscarinic acetylcholine receptor m2. *indicates kcn subunits known to regulate the function of subunits listed in the row above.

We first focused on finding candidate genes in All-Summer and VMN-Summer clusters (Figure 8A) that would contribute to known VMN neurophysiological properties (Figure 9). Neurotransmission related transcripts in All-Summer clusters included the *gap junction alpha 1* (*cx43*) that was unique to All-Summer when compared to All-Winter clusters (Table 5). Among the ion channel subunit genes upregulated in VMN-Summer clusters were *cx30*, excitatory ionotropic glutamate receptors, inhibitory glycine receptors, voltage-gated potassium channel subunits, and a *transient receptor potential cation channel subfamily M member 7* (*trpm7*) (Table 5). Interestingly, *trpm7*, which encodes a protein with both channel and kinase functions and is involved in regulating intracellular Ca^2+^ and Mg^2+^ levels, has been implicated in Guamanian ALS and Parkinson’s dementia [99].

All-Summer clusters also contained a *potassium voltage-gated channel subfamily c member 4-like* (*kcnc4*/Kv3.4) transcript (Table 5). Like the functional attributes of the *kcnc3*/Kv3.3 subunit discussed in the tissue comparisons section, potassium channels composed of KCNC4/Kv3.4 subunits could contribute to VMN’s ability to fire repetitively at high frequencies (Table 5, Figure 9). As also mentioned earlier, KCNC4/Kv3.4 currents are modulated by KCNS/Kv9 subunits, transcripts of which are highly expressed in VMN (Table 4), when coassembled in heteromeric channels [72,73]. Strikingly, in motoneurons of an ALS mouse model carrying the human *sod1* mutation, *kcnc4*/Kv3.4 was significantly downregulated relative to expression in wild type mice [100], supporting its important role in motoneuronal function.

Steroid signaling transcripts, such as *ar-a* (a different isoform than the one significantly upregulated in VMN in tissue comparisons), were found in All-Summer clusters (Table 5), indicating a summer-dependent increase in androgen sensitivity within both VMN and surrounding H. VMN’s androgen sensitivity is a shared trait with other vocal vertebrates. It has been well documented that songbird syringeal motoneurons within the nXIIts concentrate high levels of androgens [101]. Both midshipman VMN and songbird nXIIts are sexually dimorphic in size, with males having larger motoneuronal somata and neuropil volume than females [68,102,103]. Furthermore, the songbird nXIIts responds to androgen treatment by increasing the size of motoneuronal somata, synaptic density, and the number of synaptic vesicles [103,104], which could increase their ability to drive vocalizations during the breeding season. Similarly, juvenile male midshipman treated with androgens exhibit increased size of VMN somata [105]. We also found a *glucocorticoid receptor* (*nr3c1*) in VMN-Summer clusters, indicating a summer-dependent increase in glucocorticoid sensitivity specifically in the VMN (Table 5). The presence of both androgen and glucocorticoid receptors is consistent with androgen- and cortisol-induced lengthening of hindbrain vocal CPG output [33,34].

The neurotransmission and neuroendocrine mechanisms highlighted by our candidate genes could influence each other to bring about seasonal changes in VMN function and midshipman vocal behavior. For example, ultrastructure studies in songbirds and rodents have shown that estrogen and androgens can increase gap junction expression (e.g. [106-108]). Testosterone treatment in female songbirds induced an increase in the number of soma-somatic gap junctions in HVC, a telencephalic song control nucleus, correlated with an increased repetition rate and decreased variability in frequency modulation of male-like song [106]. Steroid-dependent seasonal regulation of gap junction abundance in vocal control nuclei influencing vocal behavior could also apply to midshipman fish.

#### Daily comparisons

We found nine neurotransmission transcripts within Peak-SNV clusters (Table 5) that were all unique from those found in Peak-SNH clusters. These included an isoform of c*x30*, *GABA*_*A*_*receptor subunit pi-like* (*gabrp*), and two *calcium-activated potassium channel subunit alpha-1* (*kcnma1*/Kca1.1) isoforms (Table 5, Figure 8B). While *cx30* and *gabrp* were both found in transcripts upregulated in the VMN compared to H (Table 4), they are different Trinity-predicted isoforms. Similar to between-tissue comparisons that highlighted the importance of cholinergic action in VMN, we found acetylcholine receptors, including *neuronal acetylcholine receptor beta-2-like* (*chrnb2*) in Peak-SNV clusters (Table 5, Figure 8B). Steroid signaling transcripts in Peak-SNV clusters included an *estrogen receptor alpha* (*esr1*) isoform (Table 5), shown by in-situ to be expressed in vocal motoneurons [94]. We propose that seasonal and/or daily variation in the expression levels of neurotransmission and steroid hormone related transcripts support modulation of known VMN firing properties that, in turn, translates directly into changes in vocal behavior at times of high vocal activity.

## Conclusions

The vocal motor system of midshipman fish exemplifies a simple vertebrate model with which we can identify genetic components supporting neural network function leading to a single behavior, in this case vocalization that serves a social communication function in multiple vertebrate lineages. This VMN transcriptome project provides a global view of molecular pathways responsible for neuronal function and hormone modulation in the vocal motoneurons of midshipman and vertebrate in general. We identified a suite of candidate genes whose functions underlie previously identified VMN firing properties, as well as novel candidates whose functions regulate aspects of neuronal excitability that remain to be studied in VMN (summarized in Figure 9).

The interpretive power of our results benefits directly from a large body of corroborating evidence in the midshipman model system, from synaptic ultrastructure to intracellular neurophysiology, anatomical localization of steroid signaling pathways, and hormonal modulation of VMN output and vocal behavior (Figure 9). The new results presented here guide future molecular, anatomical, and pharmacological investigations for determining how specific metabolic, hormonal, and neurotransmission pathways sculpt neurophysiological events that pattern vocal behavior.

Our findings have broad relevance to other vertebrate taxa given the highly conserved nature of vocal mechanisms, shared demands for temporal precision in sound production, and the equally widespread occurrence of seasonal and daily variation in vocal behaviors (see discussions in [3,39]). We propose that candidate genes and pathways identified here shape neurophysiological characters of motoneuronal populations driving the sound producing superfast muscles in toadfish [65], birds [4,5], bats [6], and rattlesnakes [109]. The hypothesis that fish use the same fundamental molecular machinery as other vertebrates for controlling acoustic communication is challenging but testable. Furthermore, contrasting our results to other motor systems will yield insight into the molecular machinery utilized by neuromuscular systems varying in speed, synchrony, and precision. Finally, molecular insights into motoneuronal function will also be relevant for motoneuron dysfunction, such as diseases characterized by the loss of resilience to oxidative stress.

## Methods

### Tissue collection

Midshipman fish have two male reproductive morphs: type I males that build and guard nests, acoustically court females and are the focus of this study, and type II males that sneak spawn [22,26]. We collected tissue samples from type I males at different time points in the summer reproductive and winter non-reproductive seasons (see Figure 1D). None of the fish used were actively vocalizing when sacrificed, although we cannot completely discount recently produced isolated grunting or growling behavior (see below). All procedures were approved by the Institutional Animal Care and Use Committee at Cornell University.

Reproductive fish: In May 2011, type I males were hand collected from rocky intertidal zones in northern California. Fish were then held for 1–2 days at the University of California Bodega Marine Laboratory in large outdoor tanks with artificial shelters and flow-through seawater. We are confident that the fish used in this study were not engaged in extended vocal behaviors such as humming for several reasons. First, although the fish could have been producing isolated agonistic grunts or growls in this setting, we did not observe the inflated swim bladders associated with humming (AHB unpub obs). Second, males from California do not take up residence and engage in any defense or courtship vocal behaviors until three weeks in captivity (NYF and AHB upub obs); fish used in this study were only held in large communal tanks for 1–2 days. Future transcriptome experiments using tissues from fish that were recorded and confirmed to be actively humming will provide insight into genes upregulated during vocal behavior.

The ambient light:dark cycle was approximately 14.25 h light: 9.75 h dark, with sunrise at approximately 6:00 am and sunset at 8:15 pm. The “summer night (SN)” males (n = 6) were sacrificed at the middle of the dark phase (middark), between 12:00 am and 2:00 am; and the “summer morning (SM)” males (n = 6) were sacrificed after sunrise, between 7:00 am and 9:40 am. Fish were deeply anesthetized under 0.025% benzocaine (Sigma-Aldrich, St. Louis, MO) and exsanguinated from the heart. The brain was removed after craniotomy, transected at the midbrain-hindbrain boundary, and stored in tubes containing RNAlater solution (Life Technologies, Carlsbad, CA) overnight in 4°C followed by −20°C. The samples were shipped overnight to Cornell University (Ithaca, NY) on dry ice and stored in −20°C until use.

Non-reproductive fish: Midshipman migrate offshore to deeper waters in the winter non-reproductive season [110]. In January 2011, type I males were obtained by otter trawl (R/V John H. Marin, Moss Landing Marine Laboratories) in Monterey Bay, offshore from Moss Landing, CA at depths of 115–150 meters. These fish, referred to as “winter night (WN)”, were kept under running seawater in large indoor tanks with ambient lighting through skylights, and sacrificed within 24 h of collection at middark (n = 5; 11:30 pm to 1:00 am) following the same tissue collection procedures as used for reproductive fish.

### cDNA library preparation and sequencing

The paired VMN located at the midline contain approximately 4000 homogeneous motoneurons, presynaptic input, and surrounding glial cells that can be isolated *in toto* (Figures 1C and 9) [8,45,46]). Immediately before RNA extraction, we used fine forceps and minutien pins to isolate the paired VMN from the surrounding hindbrain (H) as previously described [46]. Separation of VMN from the rest of H, which included premotor populations and other motor populations, was verified in sectioned material. Total RNA was extracted from VMN and H tissues using the Trizol reagent (Life Technologies) following the manufacturer’s protocol. RNA concentrations were quantified using a Qubit fluorometer (Life Technologies), and equal masses of RNA were pooled by tissue and collection time. Reproductive summer night (SN) and summer morning (SM) VMN and H groups (SNV, SNH, SMV, SMH) were each pooled from 6 individuals, non-reproductive winter night (WN) VMN (WNV) was pooled from 4 individuals, and winter night H (WNH) was pooled from 5 individuals (Figure 1D). Each pool contained 2 μg of total RNA. We treated the pooled RNA samples with DNase I (Ambion) to eliminate potential genomic DNA contamination.

Figure 2 provides an overview of this study’s key procedures and analyses with reference to relevant figures and tables. We closely followed the protocol of Zhong et al. [111] for strand-specific cDNA library construction, which included poly(A) RNA isolation and chemical fragmentation, first-strand cDNA synthesis, second-strand cDNA synthesis with dUTP, end-repair, dA-tailing, Y-shape barcoded adapter ligation, DNA purification and size selection. The second-strand DNA was digested with uracil DNA glycosylase to leave first-strand cDNA with differential adapters on the 5′ and 3′ ends. We then enriched our cDNA library using 14 cycles of PCR, and verified the size by agarose gel. Examining 40 ng of each cDNA library with an Agilent Bioanalyzer at Cornell’s Biotechnology Resource Center showed size peaks between 251 bp and 257 bp. Finally, 20 ng of cDNA from each sample group were combined with 20 ng from each of 4 other groups of cDNA from saccular epithelia of the inner ear into a single pool of 2000 ng pooled cDNA library; the auditory tissues contributed to a companion study [49]. The completed cDNA library was submitted for paired-end sequencing by Illumina HiSeq2000 at the Biotechnology Resource Center’s Genomics Facility.

### Transcriptome assembly and annotation

Read pairs were discarded if either read did not pass Illumina’s quality control. Adaptor sequences and low quality nucleotides were removed from the ends using Trimmomatic tools and reads less than 10 bases after trimming were dropped [112]. Over 90% of raw reads passed quality filtering and trimming, leaving 20.2 ± 2.4 million (mean ± S.D.) reads per group. Forward and reverse reads from all sample groups, including those from the saccular epithelia, were concatenated and transferred to the Blacklight system at the Pittsburgh Supercomputing Center where the full transcriptome was assembled with the Trinity software package (version r2013-02-15) [113], one of the most robust *de novo* assembly methods [114]. In order to limit occurrences of fusion transcripts we used the “jaccard_clip” option and set the “min_kmer_cov” to 2.

We used Trinity’s TransDecoder utility to discard assembled transcripts with open reading frames (ORF) of less than 50 amino acids [113]. To assess the completeness of our transcriptome, we submitted our filtered transcriptome to the CEGMA software [52]. CEGMA looks for the presence of 248 highly-conserved core eukaryotic genes (CEGs) and a larger set of 458 CEGs [52]. We used Blast2GO to annotate our assembled transcriptome based on the NCBI non-redundant protein database [53]. All transcripts were submitted to blastx using an E-value cutoff of 1e-10 via Blas2GO; those without blastx hits were then subjected to blastn a 1e-10 E-value cutoff. Mapping and annotation steps were performed with default settings (i.e., E-value cutoff = 1e-6). InterProScan and GO-Enzyme Codes were run on all transcripts.

With Trinity-supported companion programs (i.e. bowtie and RSEM) we mapped the individual reads back onto the assembled transcriptome and estimated the abundance of transcripts from each tissue group [113]. Lowly supported transcripts, accounting for less than 1% of total reads of a gene (IsoPct), were discarded. The length distribution statistics of surviving transcripts were obtained through PRINSEQ [115]. As an additional assessment of assembly quality, we submitted the final set of surviving transcripts to TransDecoder and counted the number of transcripts with complete ORFs (containing start and stop codons). The assembled transcriptome and sample group reads were submitted to the NCBI Transcriptome Shotgun Assembly and Sequence Read Archive databases under BioProject accession PRJNA269550.

Because four ear tissue groups were included in the transcriptome assembly, our analyses of the vocal system excluded transcripts with read count sums of zero across all six brain sample groups (see Figure 1D). We focused on transcripts (isoforms) rather than predicted genes (also called “gene components” by Trinity, [113]) because the single sequence available for each transcript allows straight-forward annotations and the potential to identify differential regulation of alternate isoforms of a single gene.

### VMN vs. H tissue comparisons

#### Tissue-based normalization

TMM normalization (Figure 4), used by default Trinity analysis pipeline [113], as well as several methods evaluated in [116], was insufficient in correcting the nonlinear skew observed in VMN vs H comparisons, likely due to systematic sequencing biases caused by differential tissue RNA population composition (see [56,116] for discussion). However, a cyclic loess method called fastlo [55], originally designed for microarray data, worked well to correct our nonlinear skew (Figure 4). To allow proper fastlo normalization, we added 1 to all raw counts prior to normalizing.

#### Differential expression using edgeR

For the comparison between tissues, we treated the three time points sampled as biological replicates, estimated transcript-wise dispersion values, followed by differential expression analysis with edgeR’s exact test [117]. Differentially expressed transcripts (FDR < 0.05, largely corresponding to fold change > 4; see blue lines in Figure 7A) showing upregulation in VMN or H were then subjected to downstream, GO term enrichment and KEGG pathway mapping analyses. Furthermore, we selected nine candidate genes of functional interest upregulated in VMN samples for validation by sequencing and qPCR (see below).

#### Gene category-based analyses

To gain a sense of the relative contribution of gene functional categories to VMN function (Figure 2), we extracted fastlo-normalized expression levels for transcripts (regardless of whether they were differentially expressed) that were classified under: 1) “**neurotransmission**”: ion channels and neurotransmitter receptors; 2) “**neuroendocrine**”: steroid hormone signaling genes such as receptors and biosynthetic enzymes, and thyroid hormone related genes; 3) “**neuropeptide**”: neuropeptides and their receptors, as well as peptide hormones and their receptors; 4) “**neuromodulator**”: neurochemicals other than steroids and neuropeptides that are known within this system, namely genes related to catecholamine, serotonin, and melatonin biosynthesis and signaling; 5) “**cellular respiration**”: cellular respiration-related genes; and 6) “**antioxidant**”: antioxidant enzymes and proteins. Transcript IDs and descriptions in each gene category are listed in Additional file 1: Table S4.

We compared the tissue expression levels of gene categories to assess the relative importance of each gene category for VMN vs. H (Figure 5). We first examined whether H expressed more genes by dichotomizing the data into 1 if the total tissue expression was > 0 and 0 if the total was 0 (not expressed), followed by a chi square test (Figure 5A). To compare total expression levels for each gene category, we summed expression levels across the three VMN or H sample groups for expressed genes (tissue sum > 0) (Figure 5B). We then Box-Cox transformed this dataset in order to statistically compare H vs. VMN tissue expression by performing one-way ANOVA for each gene category and setting the Bonferroni corrected alpha level at 0.008 using JMP9.0 (SAS Institute Inc., Cary, NC, USA) (Figure 5B). The non-parametric Wilcoxon rank sum test for tissue comparisons followed by Bonferroni correction produced the same results. Finally, to evaluate correlation patterns across sample groups for each gene category, we performed Spearman’s correlation for pair-wise comparisons between all six-tissue groups in R [118] (Figure 6).

### Daily and seasonal variation in gene expression

For daily and seasonal gene expression comparisons, we used Trinity’s downstream analysis pipeline with default parameters [113]. Transcripts that were differentially expressed in at least one pairwise sample comparison were identified using the Trinity-supported edgeR Bioconductor package [57], We ran the TMM normalized FPKM matrix through Trinity-supported differential expression analyses to generate hierarchically clustered heatmaps of transcripts with at least four-fold differential expression and false discovery rate (FDR) corrected P values of <0.001 (23,855 out of 77,285 transcripts) (Additional file 3). Spearman correlation matrices for pairwise sample group comparisons were also generated (Additional file 3). We generated 109 K-means clusters based on the rule of thumb for determining cluster number [119] with 23,855 differentially expressed genes from which we could extract sequence ID, annotation, and expression levels. We combined clusters exhibiting similar expression patterns of day-night and seasonal variation across sample groups. Candidate genes with functions directly related to neural transmission (e.g. ion channels) and neuromodulation (e.g. steroid hormones) were identified and considered for potential actions in sculpting VMN firing properties.

### Quantitative PCR (qPCR) validation of gene expression

We used qPCR on a subset of transcripts to confirm the accuracy of our assembled transcriptome and RSEM predicted differential transcript abundance among our sample groups. First, to validate within-tissue gene expression ratios we selected 19 transcripts chosen based on the following criteria: 1) each transcript was the only transcript in its gene component; 2) the SNH/SMH FPKM ratios of all the transcripts fell within a broad range (0.05-100); 3) the primers we chose based on the assembled transcript sequences produced only the predicted PCR product (Additional file 1: Table S6 contains gene descriptions and primer sequences). These criteria allowed for straightforward qPCR analyses from H samples, which provided more cDNA for analysis than VMN samples. Specificity of the qPCR primers was verified by sequencing at the Cornell Genomics Core facility. We selected the reference gene based on its very low coefficient of variance (0.10) in FPKM values across all sample groups and being the only transcript for its gene component. The reference gene (Blast2Go annotation “btb poz domain-containing protein kctd6”) was confirmed by qPCR to show no apparent or significant expression differences between SNH and SMN (P = 0.26). Each qPCR was performed in triplicate on the same, pooled cDNA sample produced for RNA-seq as well as no template controls. Each reaction contained 5 μl of 2x Power SYBR Green PCR Master Mix (Life Technologies, Carlsbad, CA), 1 μl each of forward and reverse primers (50 nM final concentration for the reference gene and 100 nM for target genes), 1 μl dH2O, and 2 μl of the appropriate pooled cDNA. The qPCR reaction was run on an ABI Viia7 system at the Cornell Genomics Core facility. We used the standard curve method to extrapolate copy numbers, which were normalized against our reference gene. Pearson’s correlations were calculated for qPCR and RNA-seq predicted SNH/SMH expression ratios (Figure 3D).

Next, to validate VMN vs. H tissue comparisons with qPCR, we chose nine functionally important candidate genes that were upregulated in the VMN (Additional file 1: Table S6), regardless of how many isoforms were predicted by Trinity. We used the same, pooled SMV and SMH samples that were submitted for RNAseq. SMV and SMH samples were chosen due to cDNA availability. The qPCR procedures and reference gene were the same as above for SNH/SMH comparisons. Pearson’s correlations were calculated for qPCR vs. fastlo-normalized SMV/SMH ratios, which were averaged across all isoforms for each Trinity predicted gene component (Figure 7C), as well as for qPCR vs fastlo-normalized values separately analysed for SMV and SMH (Additional file 2). Student’s paired *t* test was performed in R to compare qPCR measured SMV and SMH expression levels using log-transformed data (Figure 7D).

### Availability of supporting data

The assembled transcriptome and reads from each sample supporting the results of this article are available in the NCBI Transcriptome Shotgun Assembly and Sequence Read Archive databases under BioProject accession number [PRJNA269550].

## Additional files

Additional file 1: Table S1. GO terms enriched in transcripts upregulated in VMN. GO category notations: C = cellular component; F = molecular function; P = biological process. FDR = false discovery rate adjusted P values. GO terms related to cellular respiration are counted in column D. **Table S2.** GO terms enriched in transcripts upregulated in surrounding hindbrain (H). GO category notations: C = cellular component; F = molecular function; P = biological process. FDR = false discovery rate adjusted P values. GO terms related to cellular respiration are counted in column D. **Table S3.** KEGG pathways mapped to upregulated transcripts in VMN or H. Names and the number of sequences are listed for each KEGG pathway. **Table S4.** Functional gene category transcripts. List of sequence IDs and Blast2GO sequence descriptions for transcripts under each functional gene category. Expression levels for these transcripts are plotted in Figure 5. **Table S5.** Functionally important candidate genes upregulated in H. **Table S6.** Sequences and primers used for qPCR validation. Forward (F) and reverse (R) qPCR primer sequences are listed for each Trinity predicted isoform or gene component. Additional file 2:
**QPCR validation of RNAseq candidate gene expression.** A) SMV qPCR values (copy numbers normalized by a reference gene) for nine candidate genes are significantly correlated with RNA-seq predicted values (FPKM). B) SMH qPCR values (copy numbers normalized by a reference gene) for nine candidate genes are significantly correlated with RNA-seq predicted values (FPKM). Additional file 3:
**A) Heatmap of hierarchically clustered expression levels (median centered FPKM +1) of significantly differentially expressed transcripts based on the TMM-normalized dataset.** Each line is a transcript, and each column is a sample group. Sample groups are hierarchically clustered based on their spearman correlation coefficients. B) Heatmap of pairwise Spearman correlation coefficients. Hindbrain sample groups show higher correlation of transcript expression patterns than VMN sample groups. See Figure 1C for explanation of sample group abbreviations.

## Abbreviations

ALS: Amyotrophic lateral sclerosis
ANOVA: Analysis of variance
ATP: Adenosine triphosphate
BLAST: Basic local alignment search tool
CEGMA: Core eukaryotic genes mapping approach
CEGs: Core eukaryotic genes
CPG: Central pattern generator
FDR: False discovery rate
FPKM: Fragments per kilobase of transcript per million reads mapped
GABA: Gamma-aminobutyric acid
GO: Gene ontology
H: Surrounding hindbrain without VMN
HVC: Letter based name for a telencephalic song control nucleus in songbirds
KEGG: Kyoto encyclopedia of genes and genomes
Kv: Voltage-gated potassium channel
nXIIts: Tracheosyringeal division of the hypoglossal motor nucleus
ORFs: Open reading frames
qPCR: Quantitative PCR
RNA-seq: RNA sequencing
SMH: Reproductive summer morning hindbrain
SMV: Reproductive summer morning VMN
SNH: Reproductive summer night hindbrain
SNV: Reproductive summer night VMN
TCA: Tricarboxylic acid
TMM: Trimmed mean of M values
VMN: Vocal motor nucleus
WNH: Non-reproductive winter night hindbrain
WNV: Non-reproductive winter night VMN
