# Supplementary figures and images for: Neural transcriptome reveals molecular mechanisms for temporal control of vocalization across multiple timescales
Source: BMC Genomics. 2015 May 27;16(1):408. doi: 10.1186/s12864-015-1577-2 (PMC4446069; doi:10.1186/s12864-015-1577-2)

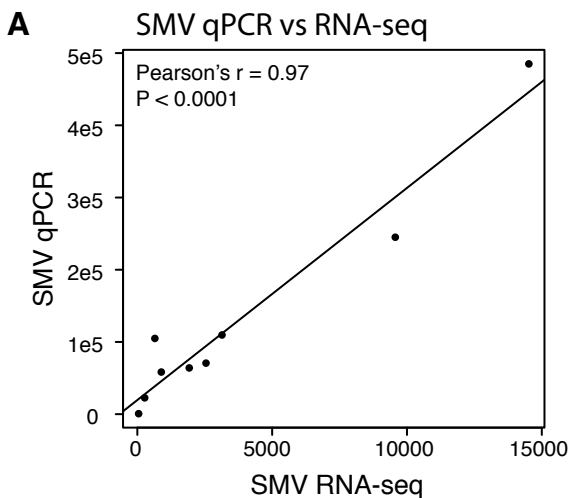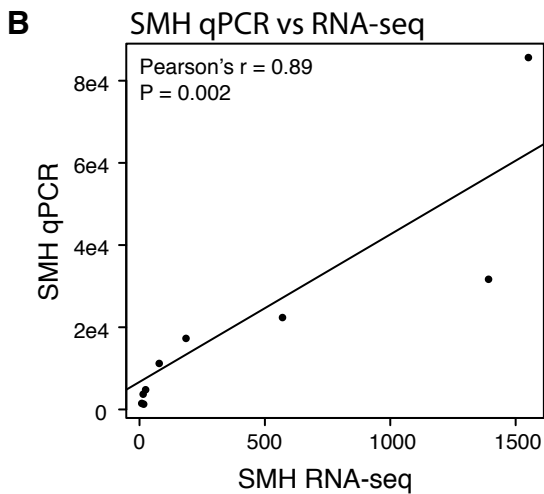

Supplement: Additional file 2: — QPCR validation of RNAseq candidate gene expression. A) SMV qPCR values (copy numbers normalized by a reference gene) for nine candidate genes are significantly correlated with RNA-seq predicted values (FPKM). B) SMH qPCR values (copy numbers normalized by a reference gene) for nine candidate genes are significantly correlated with RNA-seq predicted values (FPKM). [file 12864_2015_1577_MOESM2_ESM.pdf]

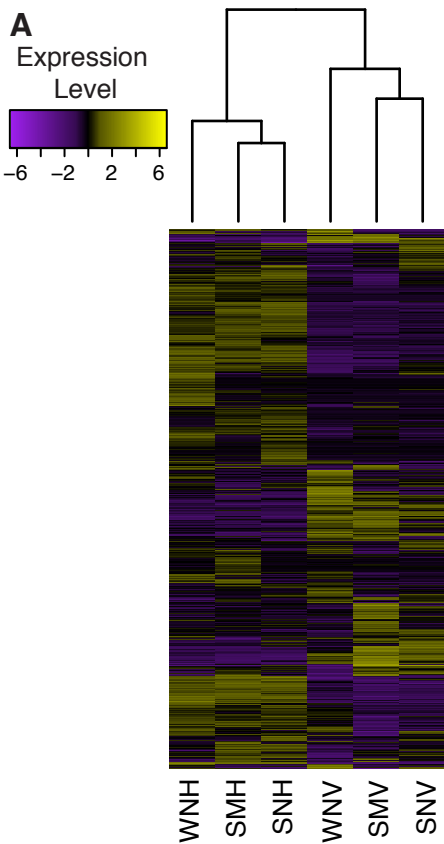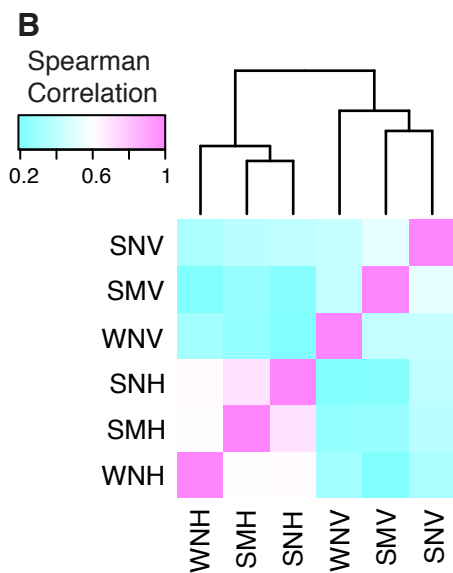

Supplement: Additional file 3: — A) Heatmap of hierarchically clustered expression levels (median centered FPKM +1) of significantly differentially expressed transcripts based on the TMM-normalized dataset. Each line is a transcript, and each column is a sample group. Sample groups are hierarchically clustered based on their spearman correlation coefficients. B) Heatmap of pairwise Spearman correlation coefficients. Hindbrain sample groups show higher correlation of transcript expression patterns than VMN sample groups. See Figure 1C for explanation of sample group abbreviations. [file 12864_2015_1577_MOESM3_ESM.pdf]
